# Treatment-Resistant Eosinophilic Spongiosis Dermatitis in a Patient With Various Comorbidities: A Case Report

**DOI:** 10.7759/cureus.94869

**Published:** 2025-10-18

**Authors:** Yao Liang, Justin Nguyen, Austin T Williams

**Affiliations:** 1 College of Medicine, Edward Via College of Osteopathic Medicine, Monroe, USA; 2 Family Medicine, Avoyelles Hospital Rural Health Clinic, Marksville, USA

**Keywords:** comorbid anxiety, dupilumab, dupixent, eosinophilic spongiosis, managing atopic dermatitis, psych-dermatological, recreational drug use, topical and systemic steroids, venous stasis dermatitis

## Abstract

Eosinophilic spongiosis is a principal term for many diseases that display a histopathology of eosinophilic infiltrates in the spongiotic epidermis. Disorders consistent with eosinophilic spongiosis include autoimmune bullous disorders, chronic eczema, and contact dermatitis. In this case report, we present a 38-year-old male with a generalized, pruritic, and erythematous rash that was diagnosed two years prior as eosinophilic spongiosis and venous stasis dermatitis based on the respective biopsies of the skin of the left elbow and of the right lower extremity. The patient reported intense pruritus that required him to wear white cotton gloves to prevent him from further scratching. His social history was notable for current tobacco use and a past history of illicit drug use. Within the two years, the patient also had numerous appointments with various primary care physicians and cardiology to address his mental health and cardiovascular symptoms such as chest pain, dyspnea, and claudication. Alongside receiving a thorough cardiovascular exam workup, the patient tried numerous treatments for his rash and the pruritus, including topical triamcinolone, tacrolimus ointment, steroid injections, hydroxyzine, amitriptyline, and, most recently, dupilumab therapy. Without much improvement with these therapies, the patient’s condition remains persistent and increasingly affects his quality of life. This case report highlights the complexity of ongoing assessment and treatment, offering guidance for the care of patients with similar presentations. Educational insights obtained from this report include maintaining broad differential diagnoses, the influence of neuropsychiatry and cardiology in dermatology, and the importance of multidisciplinary care.

## Introduction

In histology, a standard hematoxylin and eosin (H&E) stain tissue section with the presence of eosinophils within the spongiotic zones of the epidermis defines eosinophilic spongiosis (ES). Spongiotic zones are areas of edema that include wide spaces between keratinocytes with elongated intercellular bridges that give a sponge-like appearance [1]. Desmosomal spines appear stretched and inflammatory cells are distributed throughout the epidermis [1,2]. These histologic features are seen throughout various disorders that include general pruritus, inflammation caused by insect bites, allergy, atopic dermatitis, drug reaction, autoimmune bullous dermatosis, and many more [3,4].

Eosinophils invade the skin to provide host defense by releasing toxic granule proteins and reactive oxygen species, as well as communicating with other immune regulatory cells. These actions contribute to the formation of edema, blisters, and pruritus. Thus, the goal for treatment is to target and limit these functions of the eosinophils that result in clinical manifestations affecting patients. Therapeutic regimens involve resolving the patient’s pruritus and, ultimately, eosinophil-targeted therapies [5]. Antihistamines and topical corticosteroids have been used to provide itch relief, and cytokine-targeted treatment, such as dupilumab (Dupixent, Regeneron Pharmaceuticals, Sanofi), limits the inflammatory process [6].

While an exact correlation between ES and cardiovascular-associated diseases is minimally discussed in relevant studies, the disorder of venous stasis dermatitis (SD) illustrates one possible mechanism through which the two organ systems of interest influence one another. SD presents as a manifestation of chronic venous insufficiency, commonly involving the lower extremities. Erythematous and eczematous patches are present on the skin along with symptoms of pruritus, localized inflammation, and changes in the skin color and thickness [7]. While impaired wound healing caused by poor blood flow limits the need for a biopsy for SD, histology of SD has shown epidermal atrophy, extravasated erythrocytes, and prominent invasion of lymphocytes and eosinophils [7,8].

A few articles have investigated the shared presentations of chronic pruritus (CP) and psychiatric disorders [9]. Similar to cardiology, psychiatry has a prominent range of diseases that, although not directly related to eosinophilic spongiosis in literature, may be associated with dermatologic symptoms. Psychogenic CP can be classified under three frameworks: a primary dermatologic disorder with pruritus causing psychiatric conditions, a mental illness exacerbating dermatologic disorders, and a primary psychiatric disorder inducing pruritus [10,11]. Studies have examined treatments of each subtype with psychiatric therapies including serotonin reuptake inhibitors (SSRIs), serotonin and norepinephrine reuptake inhibitors (SNRIs), atypical antidepressants, tricyclic antidepressants (TCAs), antipsychotics, and anticonvulsants [10]. For instance, a systematic review by Kouwenhoven et al. on 35 studies analyzed the efficacy of oral antidepressants in patients with CP. The findings revealed a majority of cases witnessed improvement of pruritus with oral antidepressant therapies [12].

We present this case of a 38-year-old Caucasian male with comorbidities, including chronic venous insufficiency and depression, who primarily seeks relief from persistent, treatment-resistant ES dermatitis. This report aims to deepen the relationships among dermatology, cardiology, and psychiatry. In this case, predominantly exploring the diagnostic and treatment considerations is expected to contribute to a nascent outline for ES management.

## Case presentation

A 38-year-old Caucasian male with a history of generalized anxiety disorder, depression disorder, and traumatic brain injury 13 years ago, presents to his current primary care provider (PCP) for continued management of a diffuse rash found predominantly on all his extremities. The rash began three years prior and presented abruptly on various regions of the body, including the legs, face, trunk, and groin. He denied any mucosal region involvement, such as the eyes and oropharynx. The patient described symptoms of pruritus and pain in the areas affected, with significant severity indicated by his distressed state, visible excoriations, erythema, and hyperpigmentation. He also reported instances of edema in his legs and exfoliation of his skin that were not present at the visit. He denied any new detergents or skin products, along with any close contact with a similar rash. Aside from buspirone (BuSpar, Bristol-Myers Squibb), the patient was not on any other medications and did not have any known drug allergies. He had no pertinent surgical or family history. His social history was significant for current tobacco smoking of two packs per day, as well as alcohol and intravenous (IV) heroin cessation two years prior. His initial management near the time of onset involved oral hydroxyzine 25 mg and a referral to a dermatologist. Additionally, no pertinent abnormalities were discovered in a complete blood count (CBC), comprehensive metabolic panel (CMP), erythrocyte sedimentation rate (ESR), hepatitis C antibody detection, and human immunodeficiency virus 1/2 antibody detection. Antinuclear antibodies were also unrevealing. 

**Figure 1:**
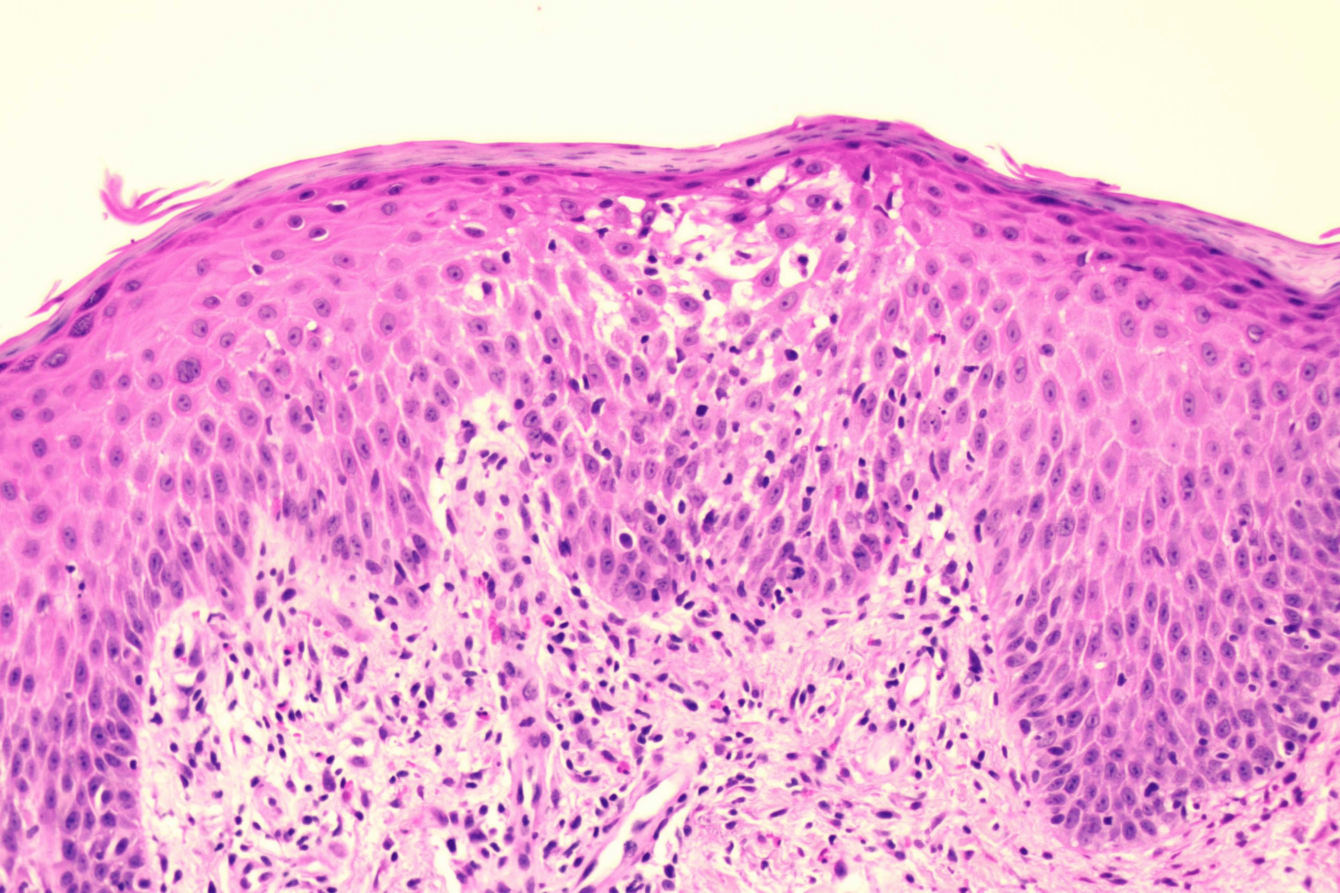
The presence of eosinophilic spongiosis. Hematoxylin and eosin stain of skin biopsy of the upper extremity, demonstrating a spongiotic zone in the center of the epidermal layer and prominent eosinophils that line the dermoepidermal junction within the superficial dermis.

The referred dermatologist performed an initial punch biopsy on the patient’s skin of the left elbow, with a resulting diagnosis of eosinophilic spongiosis with differential diagnoses of contact dermatitis and drug eruption. The pathology report described eosinophilic infiltration of the epidermis with spongiosis and negative detection of fungal organisms, which is visualized in Figure 1.

**Figure 2:**
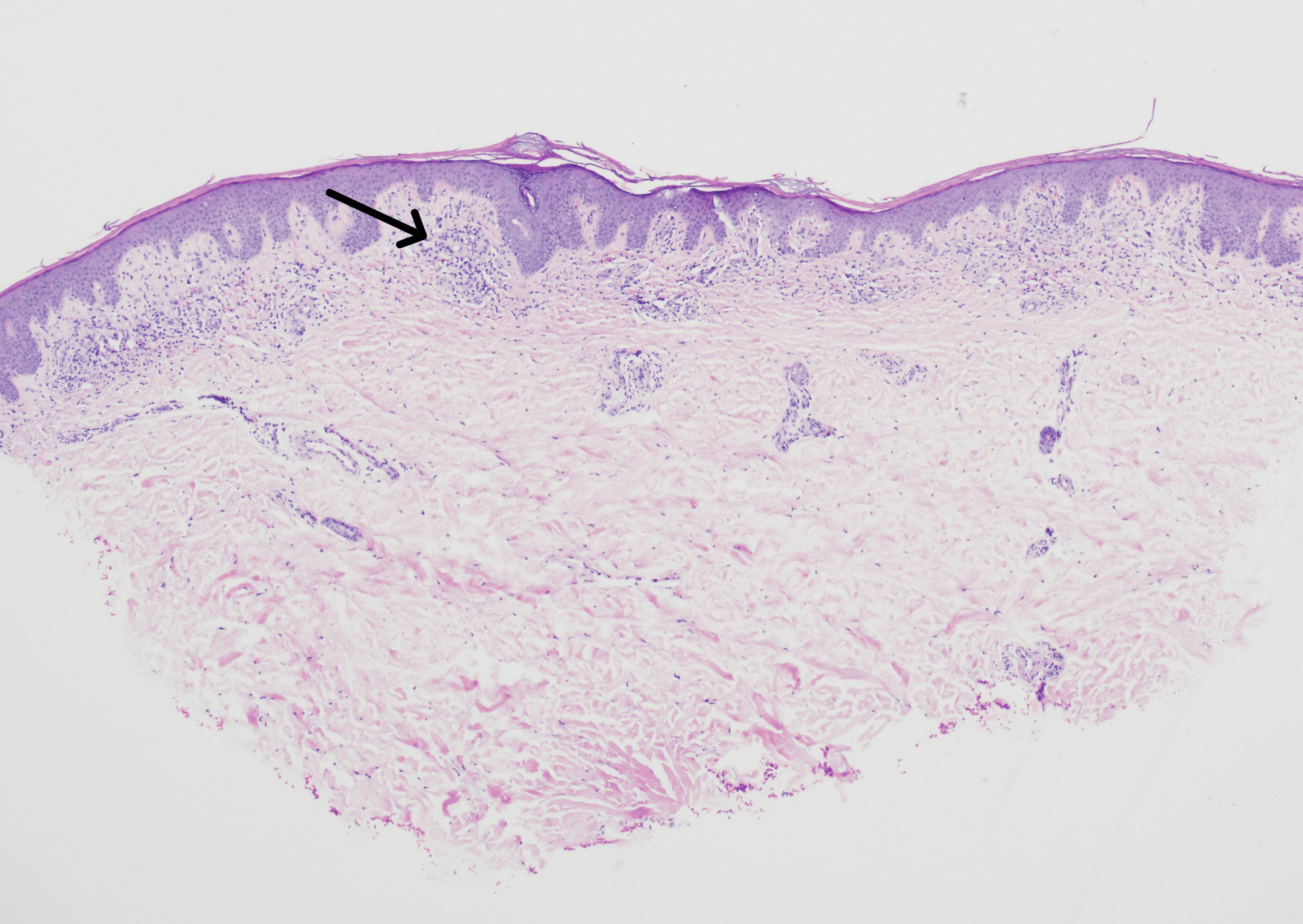
Visualization of stasis dermatitis. Hematoxylin and eosin stain of lower extremity skin biopsy, 40x low, demonstrates prominent eosinophils within the dermis, indicated by the arrow.

Although the patient reported improvement of his symptoms after receiving a corticosteroid injection and topical triamcinolone from the dermatologist, he later visited a PCP who performed a second punch biopsy, this time on his right lower extremity near the ankle. The patient received similar management, including the same laboratory evaluations and a referral to a different dermatologist. However, results from the punch biopsy revealed a different diagnosis of stasis dermatitis, as seen in Figure 2, and due to lack of insurance, the patient had discontinuous management over the next two years with visits to several different providers. Non-healing ulcers, which likely originated from traumatic excoriations, subsequently required wound care involvement.

With a different PCP, the patient was managed with topical tacrolimus 0.1% ointment. After five months, he established care with the new dermatologist, who approached the rash with a framework similar to treating atopic dermatitis, believing there to be an overlap with the previous diagnoses of stasis dermatitis and ES dermatitis. Alongside lifestyle changes, such as utilizing moisturizers and avoiding hot showers, the patient received a prescription for dupilumab (Dupixent, Regeneron Pharmaceuticals, Sanofi). The patient also presented to his present primary care clinic associated with the presentation of this case report to establish a new continuity of care.

His current treatment plan was modified to include mupirocin 2% topical ointment and sertraline 100 mg in addition to dupilumab and hydroxyzine. Medications addressing his other conditions include famotidine for gastroesophageal reflux disease and quetiapine for off-label treatment of sleep disorders. After a three-month follow-up, the patient reported no significant improvement, but he had plans to evaluate his current management with the dermatologist.

**Figure 3:**
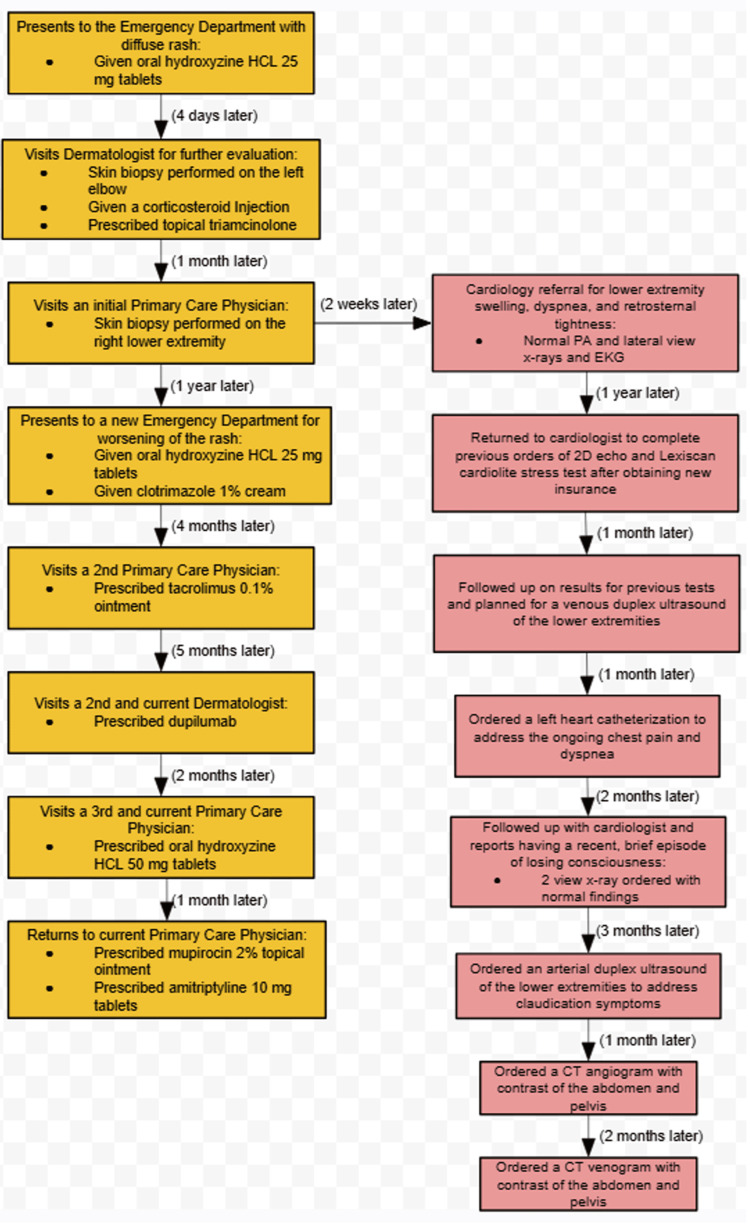
Timeline of care received. The timeline illustrates the order at which events proceeded for the management of the patient's dermatitis, described in the yellow boxes. The patient’s progression of cardiovascular diagnostic investigation is included in the red boxes. HCL: hydrochloride; EKG: electrocardiogram; 2D echo: two-dimensional echocardiogram; CT: computed tomography; PA: posteroanterior.

Notably, the patient also sought care for cardiac symptoms throughout the management of his ES, visualized by the timeline in Figure 3. The patient had been experiencing exertional tightness in the epigastric and retrosternal area, dyspnea, and intermittent lower extremity claudication. A comprehensive cardiovascular diagnostic investigation was performed throughout the years, which included, but was not limited to, a bilateral lower extremity venous duplex ultrasound, trans-thoracic echocardiogram, Lexiscan Cardiolite stress test (Astellas Pharma US, Inc., Lantheus Medical Imaging, Inc.), and left heart catheterization. While the majority of evaluations yielded normal findings, significant findings included grade 1 diastolic dysfunction of the left ventricle, mild pulmonary hypertension, reversible inferior cardiac wall perfusion defect, and venous insufficiency of the left common femoral vein with a reflux time of 2.5 seconds. These findings supported the diagnosis of stasis dermatitis and the presentation of non-healing lesions. Additionally, the patient reported to his cardiologist a moment when he experienced a loss of consciousness. His ongoing health conditions and pain worsened his depression, motivating him to seek mental health treatment.

## Discussion

The infiltration of eosinophils into the epidermis and dermis accounts for a broad differential diagnosis of inflammatory skin diseases, but eosinophilic spongiosis is distinctly defined as the presence of eosinophils within spongiotic epithelium [4]. ES has been commonly reported in pemphigus vulgaris, pemphigus foliaceus, spongiotic dermatitis, and eczematous dermatitis. Additionally, the histological findings of ES have been reported in cases of secondary syphilis [13]. Certain viral illnesses have presented with spongiotic reactions that were obscured by other pathological structure changes, including acantholysis: a process that differs from spongiosis with the presence of rounded keratinocytes, destroyed desmosomal spines, and the absence of inflammatory epidermal infiltrates [1,2]. Further investigation is required to better understand the significance of eosinophilic infiltrates in some diseases, such as syphilis [13]. However, this extensive list of associated and differential diagnoses highlights the importance of performing a comprehensive history and physical examination to best incorporate clinical pathology [3]. In our case report, the patient’s pertinent social history and ongoing medical conditions influenced his initial laboratory examinations, such as testing for IV-drug-related infections known to present with a rash. Although syphilis and current use of other recreational drugs were not examined in our patient’s serology and urine, respectively, these factors should be considered in a clinician’s evaluation for unspecified rashes similar to ES.

Examining the presence of epidermal and dermal remodeling is necessary in following an analytic algorithm for reaching a definitive diagnosis and treatment plan. Morais et al. share a figure detailing a heuristic approach for ES. Subsequent investigation of the type of intercellular structures determines the next course of action. For example, histological sections demonstrating acantholysis will require direct immunofluorescence (DIF) studies, the gold standard examination for autoimmune bullous dermatosis, to distinguish eczema and pemphigus [3]. DIF studies were not pursued in our patient’s case based on the clinician’s judgment. The clinical presentation of the rash lacked features suggestive of an autoimmune bullous dermatosis, such as blisters and mucosal involvement [3]. Through incorporating knowledge of the patient’s clinical presentation and histopathology, clinicians in this case formulated the plan to address the diagnosis and treatment of our patient’s condition in the frame of eczematous dermatitis. The pathologist who studied the biopsy of the left elbow stated the possibility of immunofluorescence studies in the case of significant clinical concerns for urticarial bullous pemphigoid. It is also important to consider the inherent limitations regarding the collection and examination of the skin specimen. Performing a punch biopsy after delivering a corticosteroid injection, sampling from different extremities, and collecting a shallow 3 mm punch biopsy may obscure the histological findings and contribute to diagnostic uncertainty. This emphasizes the role of integrating clinical context with histopathological findings in the management of eosinophilic spongiosis-associated dermatitis.

Providing treatment with topical corticosteroids and advising our patient to avoid known irritants closely follows the standard management. Prednisolone and other systemic steroids are used by some providers to treat similar skin conditions, such as atopic dermatitis, during severe symptomatic flares. However, many dermatologists generally avoid systemic steroids due to rebound flaring upon discontinuation of the medication [14]. This adverse event, alongside the other numerous side effects, including hypertension and adrenal suppression, undermines the benefits. Therefore, our patient’s current management lacks the use of oral systemic steroids, and is ultimately leading to the addition of dupilumab, which is approved in the United States as an independent therapy for moderate-to-severe atopic dermatitis. Dupilumab is a monoclonal antibody targeting interleukin-4 and -13, two cytokines that are crucial in the inflammatory process of diseases such as atopic dermatitis and asthma. Phase 3 trials of the medication against a placebo demonstrated marked improvements in patients’ symptoms of intense itch and eczematous lesions, quantified by 47.7% of adult patients receiving Dupixent 300 mg every two weeks achieving an Eczema Area and Severity Index of 75 (75% reduction of symptoms and presentation) compared to 13.3% of those on placebo [6]. These values were recorded at week 16 of being on dupilumab. As our patient has yet to reach this length of time in treatment for improvement with dupilumab, this case is a valuable reminder that support and adjuvant therapy are important factors to include in the management outline.

The goal of our patient’s current treatment is to provide relief of his pruritus while continuing the course of Dupixent therapy, which aims to reduce inflammation. Chronic pruritus is a prominent pillar of clinical burden in numerous dermatologic disorders, including ES. CP and mental wellness share a strong relationship where each can mutually influence the other. A prolonged, heightened state of stress greatly impacts normal physiology, which includes the immune response. An overabundance of proinflammatory markers, such as interleukins and tumor necrosis factor-alpha, occurs during overstimulation of the hypothalamic-pituitary-adrenal axis. Conversely, the severity of inflammatory skin conditions with CP contributes to a rise in anxiety and depression [11]. Although a direct etiologic association remains unrevealed in our patient’s original presentation, his latest condition testifies to the significant impairment in his quality of life and mental health. Management of his depression involves sertraline, a SSRI, which has shown antipruritic effects through eventual downregulation of 5-HT3 receptors from increased serotonergic signaling in the nervous system pathway responsible for itch [10]. The replacement of sertraline with amitriptyline was also discussed with the patient, as this tricyclic antidepressant has a unique analgesic influence through adenosine A1 receptors [10,15]. Thus, psychiatric intervention is a consideration that many clinicians can optimize as concomitant treatment for CP and many associated dermatoses. 

In-depth reflection on the role of the cardiovascular system in dermatologic processes is another factor that our case aims to highlight. The burden of lower extremity venous insufficiency complicates our patient’s care. Diagnosed via the second biopsy in this case, stasis dermatitis is the product of irregular flow in the venous system and cutaneous inflammation. Pain, swelling, and hyperpigmentation are signs and symptoms often seen with stasis dermatitis [7,8]. Among the limited literature on this topic, a case report exhibited atorvastatin-associated eosinophilic spongiosis [16]. Although our patient’s medications during the timeframe of the rash did not include statins, the significant venous insufficiency of the left common femoral vein, together with other cardiac findings, suggests a possible cutaneous manifestation of underlying cardiovascular disease. Huggenberger et al. reported that dramatic vasculature changes are associated with inflammatory skin diseases, including atopic dermatitis and bullous pemphigoid, both of which share the histological feature of ES [17].

## Conclusions

In conclusion, this case report outlines the diagnostic approach and management of complex eosinophilic spongiosis that has been resistant to several forms of treatment. While eosinophilic spongiosis is more of a histological description rather than a definitive diagnosis, it has classically been confirmed with biopsy. However, a comprehensive patient history is crucial to include, as it clarifies the specific disorder that exhibits eosinophilic spongiosis. In patients with recreational drug use or medications associated with skin eruptions, a detailed evaluation is important. Careful consideration of psychiatric and cardiovascular adjuvant therapy alongside mainstay dermatological treatments requires further analysis.
